# From curriculum to practice: Embedding one health competencies in animal science education

**DOI:** 10.1016/j.onehlt.2026.101639

**Published:** 2026-10-29

**Authors:** MaryGrace Erickson, Elizabeth Karcher, Kieran G. Meade

**Affiliations:** a CSA Animal Nutrition, Dayton Ohio, USA; b College of Agricultural Sciences, Penn State University, University Park, PA, USA; c School of Agriculture and Food Science, University College Dublin, Ireland; d Conway Institute of Biomolecular and Biomedical Science, University College Dublin, Ireland; e UCD One Health Centre, University College Dublin, Ireland

**Keywords:** One health, Animal science, Competencies, Pedagogy

## Abstract

Collective action to enhance the sustainability of animal production systems is urgent. Agricultural professionals face increasingly complex and interconnected challenges, including antimicrobial resistance (AMR), zoonotic diseases, environmental degradation, and biodiversity loss, which are likely to intensify under the pressures of feeding a growing global population and adapting to climate change. In response, governments and research institutions have embraced One Health - a collaborative, cross-sectoral framework which recognizes the interdependence of environmental, animal, and human health. By integrating these domains, One Health seeks to create holistic and sustainable solutions. However, realising this ambition requires the deliberate development of competencies among graduates and professionals to translate One Health principles into meaningful action. Existing One Health competency frameworks have largely focused on medical, veterinary, and public health contexts, with limited consideration of the distinctive role of animal scientists across production and food systems - spanning nutrition, breeding, health, welfare, farm management, biosecurity, food safety and environmental stewardship. The objective of this paper therefore is to provide discipline-specific contextualisation of One Health competencies and pedagogical approaches for their integration into animal science curricula. By equipping future animal scientists with the competencies to address complex, multifactorial challenges, animal science education (and educators) can help drive the systems-level changes needed to support animal, human, and ecological well-being.

## 1 Urgent need for collective action

Agriculture stands at the heart of human civilization, feeding billions and supporting economies around the world. Global production of animal-derived foods continues to occur at an enormous scale, with approximately 374 million tonnes of meat and 985 million tonnes of milk produced in 2024 [1]. In addition, feeding an expected world-wide population of approximately 10 billion people by 2050 will require a further substantial increase in food production, and animal protein is expected to remain a major component of global diets [2], [3]. This could potentially lead to further intensification of animal production systems.

Remarkably, 20% of the entire annual global livestock production is estimated to be lost to disease, with an projected cost of US$300B (World Organisation for Animal Health, WOAH [4]). Such a sizeable loss is unsustainable from a farming economic and welfare perspective underscoring the need to strengthen efforts to reduce its impact. In addition, rising demand for animal-sourced foods may contribute to an “*infectious disease trap*”, whereby increasing production pressures heightens the risk of infectious diseases and challenges our collective ambition to develop safer food systems for both animals and humans [5].

Approximately 75% of emerging infectious diseases affecting humans are zoonotic [6] and some of the causal pathogens are predicted to be among the most likely for future pandemic spread [7]. Bovine tuberculosis has resisted eradication efforts in many countries and is also responsible for a proportion of tuberculosis cases in humans [8], a risk exacerbated by drinking unpasteurised milk. Several putative links between related but less well-known animal-adapted cattle pathogens and human diseases are also now being investigated – including between *Mycobacterium avium* subsp. *paratuberculosis* (MAP) and Crohn's disease in humans [9], which could pose serious challenges. In addition, zoonotic influenza viruses can have significant impacts on wildlife and domestic animals, while livestock can provide opportunities for viral adaptation potentially facilitating onward transmission to humans [10]. Zoonotic diseases also have a disproportionate negative impact on developing countries [11], directly exacerbating poverty levels. All zoonoses directly impact the safety and sustainability of global animal production enterprises, threaten food chain security and risk compromising human health.

The prevention and control of both zoonotic and non-zoonotic infections also has important consequences for human and animal health. The World Health Organisation (WHO) predicts almost 40 million deaths by 2050 due to the exacerbation of antimicrobial resistance (AMR) alone [12] and specifically highlights that minimisation of inappropriate antibiotic use including in farming to reduce the number of AMR deaths [13]. Estimates of antibiotic use in animal agriculture are substantial and in the absence of effective alternatives, are projected to increase further [14]. The growing prevalence of antimicrobial resistance is reducing the effectiveness of first-line antibiotics and raising concerns about a potential post-antibiotic era [15]. Therefore, selective antibiotic management and alternative solutions to disease within animal production systems are urgently required [16].

Livestock are also an integral part of global biogeochemical cycles, exerting local and global effects on air quality, water quality, and soil health [17], [18]. Changes in global nutrient cycles, particularly those present in animal diets and excreted in animal manures, have created challenges for managing nutrients from livestock production in an environmentally sustainable manner [19]. Additionally, in the context of accelerating climate change, the need to design more resilient animal and crop production systems has never been more pressing [20]. Without swift and systemic reform, the risks of undermining food security, environmental collapse, and deeper social inequalities are exacerbated [21].

## 2 The One Health paradigm: all about partnership

To ameliorate the collective societal risks, governments and research institutions have embraced the One Health framework, a collaborative, cross-sectoral approach that recognizes the deep interdependence of environmental, animal, and human health [22]. Recently, a ‘*systems-based interpretation*’ [23] for One Health approaches has been adopted by the One Health High Level Expert Panel (OHHLEP) - an advisory panel to the Food and Agriculture Organisation (FAO), WHO, WOAH and the United Nations Environment Programme (UNEP). The definition states that: “*One Health is an integrated, unifying approach that aims to sustainably balance and optimize the health of people, animals and ecosystems. It recognizes the health of humans, domestic and wild animals, plants, and the wider environment (including ecosystems) are closely linked and inter-dependent. The approach mobilises multiple sectors, disciplines and communities at varying levels of society to work together to foster well-being and tackle threats to health and ecosystems, while addressing the collective need for clean water, energy and air, safe and nutritious food, taking action on climate change, and contributing to sustainable development*” [24]. With the renewal of the Memorandum of Understanding on One Health in 2026, the FAO, the United Nations Environment Programme (UNEP), the WHO, and the WOAH endorse their commitment to advancing One Health policy implementation. This policy shift has also increasingly been reflected in national government agendas at research funding and policy levels, including in the US where the first National One Health Framework was published in 2025 [25].

It should be apparent from the magnitude and complexity of the tasks faced by global agriculture, that no one single profession can address these challenges in isolation. The WHO One Health Joint Plan of Action specifically states: “*The One Health approach requires breaking down silos between disciplines and sectors - such as public health, animal health, plant health, and the environment - to jointly prevent, detect, and respond to health threats.*” By integrating these domains, One Health seeks to create solutions that are as holistic as they are sustainable. Additionally, and specifically in reference to animal health, the *One Health Joint Plan states:* “*Animal health is not the sole responsibility of the veterinary profession. It requires contributions from professionals in public health, ecology, agriculture, wildlife, environmental science, and beyond to ensure effective disease prevention, surveillance, and control*.”

There is no doubt that the success of the One Health agenda depends upon engagement across all levels of society which critically includes the farming sector. To avoid a situation where One Health is viewed as another top-down regulatory framework, significant educational efforts are urgently needed to build grass-roots support for a One Health model of stewardship for the environment and a shared responsibility for global health. Universities function to stimulate creative, interdisciplinary education, training, scientific research and extension which will service both the current One Health reorganisation and engineer future success. The transdisciplinary nature of many One Health challenges requires that One Health professionals are proficient in knowledge, skills, behaviours and attitudes that go beyond the discipline-specific knowledge delivered via traditional training programs. Instead, a competency-based approach considers the attributes which are key to collaboration and problem-solving across multi-disciplinary fields in response to the complex issues in need of One Health action.

## 3 Pedagogical and technological innovation to incorporate common One Health competencies into animal science curricula

The call for innovation in agricultural science pedagogy is not new [26] with calls for more practical, collaborative courses and to “*catalyse knowledge sharing across disciplines, institutions and experiences*” [27]. However, the One Health programme challenges us to be more ambitious and to reach beyond the traditional disciplines and institutions of agricultural science. In the context of animal science, where students are expected to address complex issues such as animal health, food security, sustainability, and One Health, pedagogical innovation is not just helpful - it is essential [28]. As the array of challenges facing global food production systems and planetary health continues to intensify, it is critical that students of agriculture, are educated, engaged, and prepared to tackle these issues using comprehensive and systems-based approaches [29].

In the context of One Health, core competencies focus on the unique competencies that all One Health professionals should have, regardless of the specific discipline of their university studies. They are not domain-specific and but instead form a shared competency framework for understanding and operationalising One Health. Whereas core One Health competencies originated in medical and veterinary medicine schools [30], [31], it is our contention that the transfer and adoption of these core competencies into pedagogical teaching and training of agriculture students is urgent and overdue, given the widespread contribution of agricultural graduates to the safety of the global food system (Fig. 1).

**Fig. 1:**
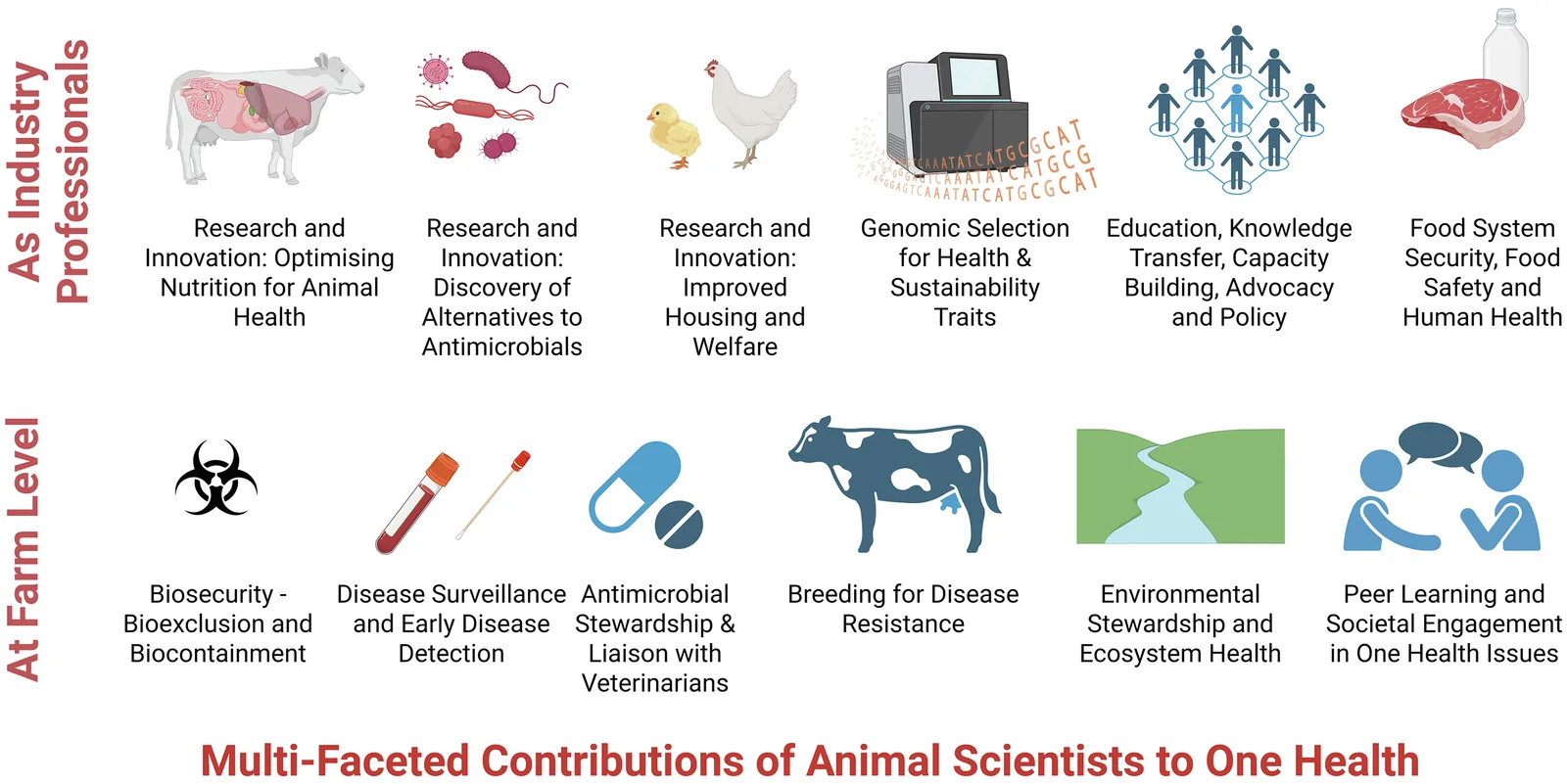
Positioned at the intersection of animal production, veterinary medicine, public health, and environmental stewardship, animal scientists are particularly well placed to implement systems-level change essential for the success of One Health initiatives. Created in BioRender. Meade, K. (2026) https://BioRender.com/k31j377.

Unifying One Health competencies have been defined previously by a number of working groups [32], and have undergone revision recently by the Network for EcoHealth and One Health (NEOH) [23]. Adapting these One Health competency domains for animal science undergraduate students is essential for preparing them to engage in interdisciplinary health challenges that span human, animal, and environmental systems. These 3 domains (Skills; Values & Attitudes and Knowledge & Awareness) comprise 9 specific core competencies are outlined in Table 1, which also shows the relevant applications within animal science.

**Table 1:** Major One Health domains and core competencies with applications relevant within animal science. Adapted from the most recent updated reference published by the Network for EcoHealth and One Health (NEOH) [23]. Competency definitions from or adapted from [23], [32], [65].

Domain	Competency & Definition	Animal Science applications
Skills	**Effective communication**. Engages effectively in respectful and reciprocal communication and partnerships with people from different backgrounds, disciplines, groups in society and sectors.	• Translate technical animal health knowledge into language understood by policymakers, producers, and the public. • Use communication tools to advocate for responsible animal husbandry, welfare, antimicrobial use, and sustainable practices.
	**Collaborative and resilient working.** Working together toward a common goal, adapting to changing circumstances and integrating diverse perspectives to achieve shared outcomes.	• Practice interdisciplinary collaboration with peers in veterinary, environmental, and public health and medical fields. • Develop communication strategies to work effectively across sectors and institutions.
	**Systems understanding.** Understanding the interconnectedness and interdependencies within complex systems, recognising how individual components, stakeholders and actions influence the wider system.	• Understand animal production as part of complex ecological, social, and economic systems. • Identify how animal health impacts and is impacted by public health, food systems, and the environment.
	**Transdisciplinarity**. Transcending disciplinary and knowledge boundaries to synthesise diverse approaches and generate new conceptual frameworks, hypotheses and strategies.	• Integrate knowledge from animal physiology, veterinary medicine, environmental science, economics and other relevant disciplines. • Engage with producers, industry, policymakers and other stakeholders (including the community) in developing solutions to complex animal production challenges.
Values & Attitudes	**Social, cultural and personal identity and inclusiveness.** Recognising and respecting differences in social, cultural and gender perspectives and ensuring that diverse perspectives and needs are considered in One Health decision-making.	• Consider how different farming systems, cultures, socioeconomic circumstances and stakeholder perspectives influence animal health, welfare and management decisions.
	**Collective learning and reflective practice.** Demonstrating reflective practice, lifelong learning and awareness of one's own assumptions, knowledge and metacognitive patterns.	• Recognise that complex animal health issues require iterative learning involving farmers, veterinarians, animal scientists and other stakeholders. • Reflect on evidence, decisions and outcomes to improve future practice.
	**Pluralism (theoretical and methodological)**. Understanding, combining and applying knowledge, theories and ideas from multiple sciences.	• Integrate biological, environmental, economic, behavioural and social perspectives when addressing animal production challenges. • Recognise and accommodate divergent stakeholder viewpoints when developing sustainable solutions.
Knowledge & Awareness	**One Health Concepts.** Understanding the principles, concepts and interconnections underpinning One Health and their application to complex health challenges at the animal-human-environment interface.	• Recognise pathways through which zoonotic diseases emerge and spread. • Understand the implications of animal management practices for antimicrobial resistance, food safety and human health.
	**Harnessing uncertainty.** Recognising, communicating and constructively working with uncertainty, ambiguity and incomplete knowledge, while adapting decisions, research approaches and actions as new evidence and perspectives emerge.	• Recognise that overcoming animal disease rarely has a single solution. • Evaluate and combine complementary approaches such as biosecurity, nutrition, breeding, vaccination and responsible antimicrobial use. • Apply risk-based approaches when evidence is incomplete.

Pedagogical innovation underpins the preparation of future professionals by ensuring that One Health education is relevant, experiential, interdisciplinary, and adaptable to real-world challenges. Interprofessional learning, defined as *learning that occurs when students from two or more professions learn with, from, and about one another to enable effective collaboration and improve outcomes*, [33] is increasingly recognised as an effective learning approach for delivering these competencies and learning outcomes [34]. In relation to AMR for example, one study reported that “*although agriculture students perceived antibiotic resistance as less threatening than Biology/pre-health students, both undergraduate groups were knowledgeable about the problem and wanted more academic education on the issu*e” [35] indicating that students are open to learning more about the issues affecting global health. Other relevant suggestions for course development include the use of shared curricula [36] and even dedicated new courses in One Health [37].

A growing body of literature demonstrates the value of diverse pedagogical approaches in developing core One Health competencies to promote critical thinking, collaboration and the application of knowledge to real-world challenges [38], [39]. A review of such innovations is beyond the scope of this paper, but selected and relevant potential approaches for both activities and assessments to optimally foster the development of each competency or learning approach in animal science are listed in Table 2. It is recognised however, that effectively incorporating strategies which adopt experiential learning approaches, including problem-based learning, into animal science curricula can be challenging, particularly due to large class sizes, time constraints and limited resources [40], [41]. In addition, other researchers highlight the challenge of assessing long-term learning outcomes [38] – specifically on whether implementation is associated with durable changes in professional behaviour or collaborative practice, particularly when students subsequently enter complex workplaces. Relevantly, low-cost options, such as field trips have been tried and found to contribute to relevant learning outcomes in relation to One Health teaching for veterinary students [42]. Another lower labour route is for educators to harness computer technologies to achieve relevant learning outcomes [43]. Artificial intelligence is being utilised in medical and veterinary medical education [44] and students need to be prepared for the revolution that it will herald in agriculture too, particularly in relation to animal health [45], [46].

**Table 2:** Selected pedagogical approaches and learning methods as well as appropriate learning activities and assessment strategies to underpin core One Health competency development in the animal sciences.

Core pedagogical approach/learning method	Description	Example learning activity	Assessment approach	Supporting reference(s)
Problem and case-based learning	Learning organised around a problem.	Investigate a complex disease outbreak involving livestock, wildlife and human health. Students identify interacting biological, environmental and socioeconomic factors and develop a response.	Group One Health problem analysis and evidence-based response plan. Students present a case from various stakeholder perspectives. Written case analysis or defended decision-making exercise.	[66]
Project and design-based learning	Learning organised around a project.	Develop a farm-level herd-health intervention addressing antimicrobial use, biosecurity or sustainability.	Multidisciplinary project proposal, stakeholder presentation and reflective report. Can involve prototype, feedback and design rationale.	[67], [68]
Experiential and reflective learning (including field visits)	Learning through experience and reflection.	Field visit to a farm, veterinary practice, food-processing facility or environmental monitoring site. Reflect on assumptions, disciplinary perspectives and decision-making during a One Health case or project.	Structured field report linking observations (e.g. using standardised evaluation checklist) to One Health principles and stakeholder perspectives. Reflective portfolio, learning journal or structured critical reflection.	[42]
Collaborative and Interprofessional learning	Learning organised around group work which integrates knowledge from different disciplines and/or professions.	Students from animal science, veterinary, environmental, food or social science backgrounds jointly analyse a One Health challenge.	Integrated multidisciplinary solution and individual reflection. Could be team based.	[34]
Interdisciplinary and transdisciplinary learning	Learning derived across academic disciplines and with stakeholders.	Interdisciplinary student teams analyse a livestock AMR case, integrating animal, veterinary and environmental perspectives and stakeholder knowledge.	Group project to develop a One Health response. Individual reflection with peer/stakeholder assessment.	[38]
Role-play & simulation	Learning through adopting stakeholder positions.	Simulated outbreak response involving farmers, veterinarians, public health officials, policymakers and industry.	Performance in simulation plus individual debrief and reflection. Prepare policy brief or reports for various stakeholders including the public.	[69]

## 4 The evolving role and identity of the Animal Scientist

The discipline of animal science has historically focused on the animal or animal production system as the subject of study. However, many undergraduate students of agriculture are current and future farmers and as such have an often under-appreciated role in the protection of the food chain and as custodians of the rural ecosystem. Moving toward a One Health framing requires a shift toward consideration of the wider systems and contexts that shape animal, human and environmental health. In his 1977 book, Wendell Berry made the prescient claim that “*The soil is the great connector of lives, the source and destination of all.*” [47] arguing that human life, culture, and the economy are inseparable from the land and that soil is not just a resource, but a living system that underpins all life and community.

Livestock species now dominate the mammalian biomass on Earth [48], and FAO/OECD data indicate that almost 90% of global meat production is derived from just three species: chickens, pigs and cattle [49]. This scale and concentration of livestock production underscore the critical and expansive role of farmers and animal scientists in global food systems, with responsibilities extending across animal production and welfare to food safety, disease prevention and the protection of environmental and public health.

Integrating One Health competencies into education and training programs is expected to better equip students with the diverse skills needed to address complex agricultural and societal challenges. Systems thinking is now recognised as critical across disciplines, including in animal science [50]. It is of direct relevance to the interactions between the microbiome and the immune system, for example, to more effectively treat animal diseases [51]. A similar conceptual framework is critical to understand the emergence of disease within an ecosystem model framework [52], [53]. and to maximise the utility of such data in finding solutions, students need advanced training in systems level skills and in this context pedagogical innovation is required. Relevant examples include the design of field school experiences which bridge the gap between One Health theory and practice in real-world settings [54], and others which incorporate advanced understanding of other equally relevant components including animal welfare [55] and One Health economics [56].

At a more rudimentary but no less important level, there is a recognised need to improve communication skills in undergraduate students of agriculture [57], [58], and part of the reward for incorporating core One Health competencies will be through the development of a common language across disciplines [59] which will promote improved collaboration, policy engagement [60], advocacy, and ultimately societal change. The future animal science graduate, better equipped with One Health competencies will engage more readily with veterinary and allied health professionals to tackle the complexity of animal health issues. In addition, via their employment across the sector including in government regulatory roles, graduates will be positioned to encourage transdisciplinary collaborations to effect positive systems change. Finally, across society at farming level and across rural communities, capable graduates will help embed One Health principles and engender support at grass-roots level which will secure future successes.

## 5 Conclusions

We have a rapidly closing window to reform food systems before systemic risks escalate. Urgent, coordinated action by governments, farmers, corporations, and consumers can chart a path toward resilient, equitable, and regenerative agriculture [20], [61]. Pandemic preparedness is now attracting significant attention which will require many more sectoral interests to identify with and take responsibility for One Health actions to reduce emerging risks and improve future responses [62]. Universities have a key responsibility here to urgently strengthen and formalise One Health competencies requiring educational approaches that deliberately create opportunities for interdisciplinary learning, collaboration and the application of knowledge to complex real-world problems [63]. Positioned at the intersection of animal production, veterinary medicine, public health, and environmental stewardship, animal scientists are ideally positioned to engage with complex, interconnected challenges [64] and to ultimately implement systems-level change so critical to effective One Health operationalisation.

## Declaration of generative AI and AI-assisted technologies in the manuscript preparation process

Statement: During the preparation of this work the author KM used ChatGPT in order to explore relevant competencies and pedagogical concepts. After using this tool/service, KM reviewed and edited the content as needed and takes full responsibility for the content of the published article.

## CRediT authorship contribution statement

**MaryGrace Erickson:** Writing – review & editing. **Elizabeth Karcher:** Writing – review & editing. **Kieran G. Meade:** Writing – original draft.

## Consent for publication

Not applicable.

## Ethics approval and consent to participate

Not applicable.

## Funding

No funding was obtained in support of this research.

## Declaration of competing interest

The authors declare that they have no known competing financial interests or personal relationships that could have appeared to influence the work reported in this paper.

## Data Availability

No data was used for the research described in the article.
